# A comparison of home care quality indicator rates in two Canadian provinces

**DOI:** 10.1186/1472-6963-14-37

**Published:** 2014-01-25

**Authors:** Amanda M Mofina, Dawn M Guthrie

**Affiliations:** 1 Department of Kinesiology and Physical Education, Wilfrid Laurier University, 75 University Ave. W, Waterloo, ON N2L 3C5, Canada

**Keywords:** Quality indicators, Home care, Standardized assessment, RAI-HC

## Abstract

**Background:**

Home care is becoming an increasingly vital sector in the health care system yet very little is known about the characteristics of home care clients and the quality of care provided in Canada. We describe these clients and evaluate home care quality indicator rates in two regions.

**Methods:**

A cross-sectional analysis of assessments completed for older (age 65+) home care clients in both Ontario (n = 102,504) and the Winnipeg Regional Health Authority (n = 9,250) of Manitoba, using the Resident Assessment Instrument for Home Care (RAI-HC). This assessment has been mandated for use in these two regions and the indicators are generated directly from items within the assessment. The indicators are expressed as rates of negative outcomes (e.g., falls, dehydration). Client-level risk adjustment of the indicator rates was used to enable fair comparisons between the regions.

**Results:**

Clients had a mean age of 83.2 years, the majority were female (68.6%) and the regions were very similar on these demographic characteristics. Nearly all clients (92.4%) required full assistance with instrumental activities of daily living (IADLs), approximately 35% had activities of daily living (ADL) impairments, and nearly 50% had some degree of cognitive impairment, which was higher among clients in Ontario (48.8% vs. 37.0%). The highest quality indicator rates were related to clients who had ADL/rehabilitation potential but were not receiving therapy (range: 66.8%-91.6%) and the rate of cognitive decline (65.4%-76.3%). Ontario clients had higher unadjusted rates across 18 of the 22 indicators and the unadjusted differences between the two provinces ranged from 0.6% to 28.4%. For 13 of the 19 indicators that have risk adjustment, after applying the risk adjustment methodology, the difference between the adjusted rates in the two regions was reduced.

**Conclusions:**

Home care clients in these two regions are experiencing a significant level of functional and cognitive impairment, health instability and daily pain. The quality indicators provide some important insight into variations between the two regions and can serve as an important decision-support tool for flagging potential quality issues and isolating areas for improvement.

## Background

A 50% increase in the number of individuals receiving home care has been observed in Canada within the last decade largely due to the essential role home care plays in primary care and chronic disease management [1]. Home care in Canada costs $3.4 billion annually, [2] and approximately 80% of home care clients are 65 years of age or older [3]. In the US, home care costs in 2010 were in excess of $70 billion [4], and similar to Canada, the majority of clients were older adults [5]. Based on this trend, home care could expect an increase in the volume of clients in the upcoming years with the escalation in the number of older adults with chronic conditions. Home care services will have to function both efficiently and effectively to provide optimal services to the greatest number of clients. In order to achieve this goal, quality of care should be continually assessed in order to support ongoing continuous quality improvement. However, in Canada, little is currently known about home care clients or the quality of care they receive [6-10].

Home care in Canada is publicly funded and is financed through the provincial government [1]. The provincial/territorial ministries provide funding, ensure compliance with policies and administer legislation and regulations. Home care in Canada is not standardized across all provinces and, unlike most other health care services, it is not regulated by the Canada Health Act (1984) [11].

In several US states (e.g., Michigan, New Jersey, Massachusetts, North Carolina) and in multiple regions in Canada, including Ontario and in the Winnipeg area of Manitoba (Winnipeg Regional Health Authority or WRHA), the Resident Assessment Instrument for Home Care (RAI-HC) is being used for assessing home care clients in a standardized fashion. The RAI-HC was developed by interRAI (http://www.interrai.org), an international group of researchers and clinicians who continually develop and refine standardized assessment instruments for older adults and individuals with disabilities [12]. The RAI-HC is an assessment system that includes a set of Clinical Assessment Protocols [13]. Some items within the assessment act as a link to the CAPs by identifying clients at risk of various negative outcomes such as cognitive decline, pain and falls. The CAPs are generated with computer software and there is also written documentation for each CAP that provides the assessor with guidance in terms of further assessment and development of a care plan (see Table 1 for detailed descriptions of the CAPs).

**Table 1:** **Description of clinical assessment protocols**[13]

**CAP**	**Description**
Activities of daily living/rehabilitation potential	Identify clients with the potential for improved independence in activities of daily living or self-care
Instrumental activities of daily living (IADLs)	Identify clients who could improve on IADLs (e.g., meal preparation, managing finances or medications)
Health promotion	To promote healthy behaviours such as increased physical activity and smoking cessation
Institutional risk	Identify individuals with a high risk of institutionalization and suggests ways to help them stay in the community
Communication disorders	Recognize communication issues and provide strategies to aide in effective communication between clients and others
Visual function	Evaluate clients with new or existing vision impairments
Alcohol use and hazardous drinking	Identify alcohol use that may be excessive or at a level that might put client at risk of negative outcomes
Cognition	Determine whether cognitive problems are present, either acute or long-standing
Behaviour	Identify persons with behavioral symptoms such as wandering, being verbally or physically abusive
Depression and anxiety	Assist in identifying persons who exhibit signs/symptoms of anxiety or depression and put forward potential treatment options
Elder abuse	Identify clients who are living in abusive or neglectful conditions or who are risk of being in this situation
Social function	Assist with maintaining or restoring meaningful social connections
Cardio-respiratory	Identify problems of the cardiovascular or respiratory systems that require intervention by a medical professional
Dehydration	Alert the professional to clients experiencing dehydration or clients who are at risk of this outcome
Falls	Identify recent fall occurrences and if the client is at risk of falling in the future
Nutrition	Detection of malnutrition and clients who may be at risk for poor nutrition
Oral health	Detect oral health problems that may be leading to outcomes such as pain, difficulty with eating/talking, poor nutrition or a lack of enjoyment of food
Pain	Identify clients with pain that may result in impairments in everyday function
Pressure ulcers	Assist in identifying clients who have pressure ulcers or skin breakdown or who are at risk of this outcome
Skin and foot conditions	Identify persons who have skin conditions or issues related to the health of their feet (e.g., corns, bunions, fungi) or who are at risk of developing them
Adherence	Identify whether issues of non-adherence to suggested treatments/therapies is present
Brittle supports	Identify families who may be having difficulty continuing in their caregiving role
Medication management	Compile a list of all medications (both prescription and over-the-counter) being taken and asses if the client is at risk of inappropriate drug therapy
Palliative care	Evaluate whether a palliative approach to care is warranted
Preventative health care measures: immunization and screening	Identify clients who have not received preventative strategies such as blood pressure check, vaccinations
Psychotropic drugs	Identify individuals taking psychotropic drugs and who might benefit a review of their medications by a physician
Reduction of formal services	Evaluate the formal services being delivered currently and determine if the current formal services are beneficial or could be modified
Environmental assessment	Identify conditions in the home that may be hazardous and compromise client safety
Bowel management	Evaluate problems in bowel functioning as well as gastrointestinal issues
Urinary incontinence and indwelling catheter	To identify when incontinence is present or a catheter is being used and address the underlying causes of incontinence

Although the RAI-HC was developed primarily to guide care planning, items within the assessment can also be used to generate a set of 22 home care quality indicators (HCQIs) [7]. These indicators are generated using computer software and represent *potential* quality issues, but should not be thought of as definitive measures of quality. The indicators were developed to act as a flag, such that high rates on the indicators would necessitate the need for further exploration by home care staff to better understand what factors were influencing the rate (e.g., processes of care). Among the 22 HCQIs, 16 are prevalence-based and the remaining six are incidence-based indicators of quality. Prevalence-based indicators allow for examination of quality at a single point in time. Incidence-based measures, however, reflect client changes in status since they incorporate data at two points in time and represent new instances of the quality issue [7]. The set of 22 HCQIs can highlight potential quality issues and can also be used to monitor changes over time resulting from quality improvement interventions implemented by home care organizations.

For 19 of the indicators, client-level risk adjusters have been proposed to allow for fair comparisons between providers [7]. Clients experiencing poorer health or co-morbidity typically develop more complications and, as a result, have worse outcomes. Risk adjustment is a statistical technique used to attempt to control for the differences in the level of risk between clients residing in different regions. Case-mix differences across regions can lead to inaccurate perceptions of quality and risk adjustment is a method used to attempt to “level the playing field” across different regions [14]. For example, for the prevalence of weight loss indicator, two risk adjusters have been suggested, namely, more severe ADL impairment and a diagnosis of cancer. To date, very few studies have been completed to examine the influence of risk adjustment [8] or to explore how these indicators vary with characteristics of the provider organizations [15].

There is the potential for the HCQIs to be used for a variety of purposes and to reach multiple target audiences. For example, the HCQIs can be used for reports to guide internal quality improvement [7]. Currently in Ontario, several of the HCQIs are being used by Health Quality Ontario, which is funded by the provincial government, to provide public information on home care providers across the province [16]. Although this is a fairly new initiative, these reports have the potential to benefit both the client and home care organization by highlighting providers where quality issues may be of concern and also by identifying top performers whose policies and practices could serve as important models for other providers.

To date, the RAI-HC has been mandated for use in multiple regions across Canada (e.g., Ontario, British Columbia, Yukon Territory) yet little Canadian evidence exists that describes home care clients and rates of quality issues by utilizing standardized data available from this assessment [7,17]. The current study was designed to address this gap by providing a comprehensive description of home care clients in these two regions and also examines the HCQI rates between these regions and how the risk adjustment process influenced these rates.

## Methods

For the current study, data from Ontario and the WRHA were chosen based on several criteria including the fact that they are two of the four provinces who have submitted RAI-HC data to a national data warehouse (held by the Canadian Institute for Health Information), they have submitted a large number of assessments and they have the longest history of RAI-HC implementation in Canada. Trained health professionals (typically nurses) collected the data as part of normal practice using the RAI-HC on laptop computers. The RAI-HC was administered on admission and re-assessments were completed every 6 months or upon significant clinical change. The completed assessments were uploaded into their local database and the two regions then submitted the data to the Canadian Institute for Health Information. This project represented secondary analysis of anonymized RAI-HC data from 2006–2010. As such, institutional ethics review was not required. These data were accessed free of charge through the Graduate Student Data Access Program (the first author was a graduate student at the time this project began). The application to request access to these data involved a description of the current project and required data, intentions regarding dissemination of results as well as computer security confirmation.

From the original dataset (n = 785,169) the sample was restricted to clients who were at least 65 years of age, had at least two assessments (for the incidence-based HCQIs) or those clients with one assessment that was not their initial assessment (for prevalence-based HCQIs). Only re-assessments were used in calculating the prevalence indicators since the home care providers would not have had time to implement a care plan for these clients and this could negatively affect the HCQI rates [7].

Additionally, the Ontario sample was further restricted to assessments completed in 2010 (n = 102,554), the most recent year of data available from the Canadian Institute for Health Information. The final dataset (n = 174,112 assessments) included 9,250 unique assessments from clients from Manitoba, specifically, from the WRHA since this was the only health region submitting data to the Canadian Institute for Health Information. To maximize the sample size in the WRHA, the most recent data available from the Canadian Institute for Health Information were used (2006–2007). We did not have access to Ontario data for 2006–2007 to align with the Winnipeg sample, and therefore chose instead to use the most recent data available for Ontario (2010). Where multiple assessments were available (9.1% of assessments in Winnipeg and 14.6% in Ontario), the most recent assessment was kept for analysis, to better reflect each client’s current health status.

Embedded within the RAI-HC are a number of health sub-scales that are generated directly from items within the assessment that have been previously validated. For example, the Depression Rating Scale summarizes seven items and has been shown to be a valid indicator of clinically relevant signs/symptoms of depression [18,19]. The Cognitive Performance Scale has been validated against the Mini-Mental State Examination [20,21]. The Activities of Daily Living Self-performance Hierarchy Scale is a valid and reliable measure of the client’s level of independence across four ADL items. It ranges from zero to six, with a higher score indicating a greater level of dependence on others [22]. The Changes in Health End-stage Disease Signs and Symptoms scale measures health instability and higher scores have been shown to be related to an increased risk of mortality [23,24]. The Pain Scale is based on two pain items related to the frequency and severity of daily pain. It can range from 0 (no pain) to 3 (severe daily pain) and has shown to be highly predictive of pain on the Visual Analogue Scale in nursing home residents [25].

T-tests or chi-square analyses were used, as appropriate, to compare the provinces on demographic characteristics (e.g., sex), the health sub-scales and the CAPs. An absolute difference between regions of at least 10% was used to define differences that were clinically relevant. Given the large sample size, very small and likely meaningless differences would be statistically significant with an alpha level of 0.05.

The HCQIs were all calculated based on items within the RAI-HC and are typically expressed as rates of issues to be avoided in the home care population [7]. Each HCQI is defined by both a numerator and a denominator, with the denominator used to identify the population at risk. For example, the inadequate pain control HCQI is calculated by dividing the number of clients who experience pain that is not adequately controlled by their medications (numerator) by the number of clients who are experiencing pain (denominator). The client-level risk adjusters were applied as outlined by Hirdes et al. [7]. All analyses were completed using SAS software v. 9.2 (SAS Institute Inc., Cary, NC).

## Results

The mean age of the sample was 83.2 years (sd = 7.6), the majority were female (68.6%) and approximately 60% of clients were divorced, separated or were widowed (Table 2). In the WRHA, a higher proportion of clients had some high school education or graduated from high school (11.7% difference). Overall, the two provinces were very similar with respect to demographic characteristics.

**Table 2:** Demographic characteristics comparing Ontario and the Winnipeg Regional Health Authority (n = 111,804)

	**Overall (n = 111,804)**	**Ontario (n = 102,554)**	**WRHA (n = 9,250)**	**p value**
	**% (n)**			
**Age**				
Mean (SD)	83.2 (7.6)	83.2 (7.6)	83.4 (7.4)	0.003
65–74	15.7 (17,560)	15.8 (16,224)	14.4 (1,336)	0.0007
75–84	40.2 (44,893)	40.0 (41,058)	41.5 (3,835)	
85+	44.1 (49,351)	44.1 (45,2721)	44.1 (4,079)	
**Sex**				
Female	68.6 (76,738)	68.3 (70,040)	72.4 (6,698)	<0.0001
**Marital status**				
Never married	4.6 (5,146)	4.3 (4,428)	7.8 (718)	<0.0001
Married	34.7 (38,844)	35.3 (36,217)	28.4 (2,627)	
Widowed/ separated/divorced	59.9 (66,974)	59.6 (61,139)	63.1 (5,835)	
Other	0.8 (840)	0.8 (770)	0.8 (70)	
**Highest level of education**				
No schooling	2.0 (2,078)	2.1 (1,976)	1.1 (102)	<0.0001
Eighth grade or less	23.8 (24,316)	23.8 (22,028)	24.7 (2,288)	
Some high school/high school graduate	33.2 (33,899)	32.2 (29,841)	43.9 (4,058)	
Post-Secondary education	21.5 (21,943)	21.6 (19,993)	21.1 (1,950)	
Unknown	19.4 (19,761)	20.4 (18,909)	9.2 (852)	

The majority of home care clients (92.4%) required full assistance with instrumental activities of daily living (IADLs). Approximately 35% of the sample experienced ADL impairment, and nearly 50% of the clients had some degree of cognitive impairment. The two regions were very similar on the health sub-scales with the exception of the Cognitive Performance Scale in which the rate of cognitive impairment was 11.8% higher in Ontario (Table 3).

**Table 3:** Health and well-being comparing clients from Ontario and the WRHA (n = 111,804)

**Health sub-scales**	**All (n = 111,804)**	**Ontario (n = 102,554)**	**WRHA (n = 9,250)**	**p value**
**% (n)**				
**IADL difficulty scale**				
Independent or Some Help (0,1)	7.6 (8,523)	7.4 (7,560)	10.4 (963)	<0.0001
Full Help (2+)	92.4 (103,281)	92.6 (94,994)	89.6 (8,287)	
**ADL self-performance hierarchy scale**				
No impairment (0,1)	65.2 (72,910)	64.6 (66,208)	72.5 (6,702)	<0.0001
Impairment (2+)	34.8 (38,894)	35.4 (36,346)	27.6 (2,548)	
**Depression rating scale**				
No signs/symptoms of depression (0,1, 2)	84.3 (94,270)	83.8 (85,898)	90.5 (8,372)	<0.0001
Signs and symptoms of depression (3+)	15.7 (17,534)	16.2 (16,656)	9.5 (878)	
**Cognitive performance scale**				
Intact or mild impairment(0,1)	52.2 (58,304)	51.2 (52,479)	63.0 (5,825)	<0.0001
Cognitive impairment (2+)	47.8 (53,481)	48.8 (50,056)	37.0 (3,425)	
**Pain scale**				
No pain/less than daily pain (0,1)	45.6 (50,967)	45.3 (46,500)	48.3 (4,467)	<0.0001
Daily pain/severe (2+)	54.4 (60,832)	54.7 (56,049)	51.7 (4,783)	
**CHESS scale†**				
Low level of health instability (0,1)	63.0 (70,371)	62.3 (63,883)	71.1 (6,488)	<0.0001
Mild/severe level of health instability (2+)	37.0 (41,315)	37.7 (38,671)	29.0 (2,644)	

†CHESS = Changes in Health, End-stage disease, Signs and Symptoms Scale [23].

The top five most frequent CAPs present overall were: preventive health measures (89.6%), IADL (81.6%), pain (68.1%), communication (64.2%) and health promotion (55.7%). The top three most frequent CAPs were identical in the two regions (Table 4). Ontario clients had higher absolute rates for 80% of the CAPs, indicating a higher level of current or future risk for these clinical issues. The opposite was seen for the brittle support CAP, which was 12.5% higher in the WRHA. This CAP identifies caregivers who are becoming stressed and at risk of not being able to continue in their caregiving role. Given that Ontario clients had higher levels of impairment generally, this finding was counterintuitive. However, we observed that clients in the WRHA were roughly 17% *less* likely to have a secondary informal caregiver (57.9% vs. 74.7%) implying that their primary caregiver was likely shouldering more of the overall caregiver responsibilities and this likely explains why the CAP rate was higher in the WRHA.

**Table 4:** Rates for the clinical assessment protocols comparing Ontario and the WRHA (n = 111,804)

**CAPs**	**Overall (n = 111,804)**	**Ontario (n = 102,554)**	**WRHA (n = 9,250)**	**p value**
**% (n)**				
***Preventative health measures: immunization and screening***				
Present	89.6 (91,374)	90.0 (83,466)	85.5 (7,908)	<0.0001
Not present	10.4 (10,626)	10.0 (9,284)	14.5 (1,342)	
***IADL***				
Present	81.6 (83,245)	82.4 (76,396)	74.0 (6,849)	<0.0001
Not present	18.4 (18,755)	17.6 (16,354)	26.0 (2,401)	
***Pain***				
Present	68.1 (76,085)	68.2 (69,898)	66.9 (6,187)	0.01
Not present	32.0 (35,719)	31.8 (32,656)	33.1 (3,063)	
***Communication disorder***				
Present	64.2 (71,753)	64.8 (66,410)	57.8 (5,343)	<0.0001
Not present	35.8 (40,051)	35.2 (36,144)	42.2 (3,907)	
***Health promotion***				
Present	55.7 (98,761)	55.2 (56,636)	60.7 (5,612)	<0.0001
Not present	44.3(75,351)	44.8 (45,918)	39.3 (3,638)	
***Cognition***				
Present	54.3 (60,707)	55.3 (56,673)	43.6 (4034)	<0.0001
Not present	45.7 (51,097)	44.7 (45,881)	56.4 (5,216)	
***Urinary incontinence and indwelling catheter***				
Present	54.1 (60,491)	55.0 (56,357)	44.7 (4,134)	<0.0001
Not present	45.9 (51,313)	45.1 (46,197)	55.3 (5,116)	
***Falls***				
Present	49.7 (55,527)	50.9 (52,235)	35.6 (3,292)	<0.0001
Not present	50.3 (56,277)	49.1 (50,319)	64.4 (5,958)	
***ADL/rehabilitation***				
Present	46.0 (51,385)	47.2 (48,356)	32.8 (3,029)	<0.0001
Not present	54.0 (60,419)	52.9 (54,198)	67.3 (6,221)	
***Medication management***				
Present	44.0 (49,220)	44.9 (46,051)	34.3 (3,169)	<0.0001
Not present	56.0 (62,584)	55.1 (56,503)	65.7 (6,081)	
***Psychotropic drugs***				
Present	37.6 (42,070)	38.8 (39,788)	24.7 (2,282)	<0.0001
Not present	62.4 (69,734)	61.2 (62,766)	75.3 (6,968)	
***Cardio-respiratory***				
Present	35.2 (39,332)	35.2 (36,080)	35.2 (3,252)	1.0
Not present	64.8 (72,472)	64.8 (66,474)	64.8 (5,998)	
***Visual function***				
Present	34.7 (38,837)	34.9 (35,783)	33.0 (3,054)	0.0003
Not present	65.3 (72,967)	65.1 (66,771)	67.0 (6,196)	
***Skin and foot conditions***				
Present	33.9 (37,884)	34.6 (35,490)	25.9 (2,394)	<0.0001
Not present	66.1 (73,920)	65.4 (67,064)	74.1 (6,856)	
***Pressure ulcers***				
Present	28.3 (31,649)	29.2 (29,934)	18.5 (1,715)	<0.0001
Not present	71.7 (80,155)	70.8 (72,620)	81.5 (7,535)	
***Depression and anxiety***				
Present	26.2 (29,274)	27.0 (27,645)	17.6 (1,629)	<0.0001
Not present	73.8 (82,530)	73.0 (74,909)	83.0 (7,621)	
***Institutional risk***				
Present	25.3 (28,315)	26.0 (26,709)	17.4 (1606)	<0.0001
Not present	74.7 (83,489)	74.0 (75,845)	82.6 (7,644)	
***Bowel management***				
Present	23.2 (25,965)	24.0 (24,558)	15.2 (1,407)	<0.0001
Not present	76.8 (85,839)	76.1 (77,996)	84.8 (7,843)	
***Oral health***				
Present	19.1 (21,348)	19.9 (20,374)	10.5 (974)	<0.0001
Not present	81.9 (90,456)	80.1 (82,180)	89.5 (8,276)	
***Nutrition***				
Present	19.0 (32,321)	19.3 (19,832)	15.4 (1,426)	<0.0001
Not present	81.0 (141,791)	80.7 (82,722)	84.6 (7,824)	
***Social function***				
Present	18.1 (20,193)	18.0 (18,460)	18.7 (1,733)	0.08
Not present	81.9 (91,611)	82.0 (84,094)	81.3 (7517)	
***Brittle support***				
Present	13.1 (13,367)	12.0 (11,103)	24.5 (2,264)	<0.0001
Not present	86.9 (88,633)	88.0 (81,647)	75.5 (6,986)	
***Reduction in formal services***				
Present	10.8 (11,026)	10.7 (9,904)	12.1 (1,122)	<0.0001
Not present	89.2 (90,974)	89.3 (82,846)	87.9 (8,128)	
***Behaviour***				
Present	10.7 (11,942)	10.9 (11,177)	8.3 (765)	<0.0001
Not present	89.3 (99,862)	89.1 (91,377)	91.7 (8485)	
***Adherence to treatment and therapies***				
Present	6.7 (7,460)	6.8 (6,950)	5.5 (510)	<0.0001
Not present	93.3 (104,344)	93.2 (95,604)	94.5 (8,740)	
***Environmental assessment***				
Present	4.1 (4,626)	4.0 (4,087)	5.8 (539)	<0.0001
Not present	95.9 (107,178)	96.0 (98,467)	94.2 (8,711)	
***Dehydration***				
Present	3.6 (4,002)	3.7 (3,837)	1.8 (165)	<0.0001
Not present	96.4 (107,802)	96.2 (98,717)	98.2 (9,085)	
***Elder abuse***				
Present	2.6 (2,893)	2.8 (2,836)	0.6 (57)	<0.0001
Not present	97.4 (108,911)	97.2 (99,718)	99.4 (9,193)	
***Alcohol dependence and hazardous drinking***				
Present	1.0 (1,107)	1.0 (992)	1.2 (115)	0.01
Not present	99.0 (110,697)	99.0 (101,562)	98.8 (9,135)	
***Palliative care***				
Present	1.0 (1,092)	1.0 (1,043)	0.5 (49)	<0.0001
Not present	99.0 (110,712)	99.0 (101,511)	99.5 (9,201)	

### Home care quality indicators

The unadjusted rates for the majority (17 of the 22) of the HCQIs were higher in Ontario than the WRHA (Table 5). The most prevalent HCQI in both regions was ADL/rehabilitation potential and not receiving therapy, followed by hospitalization in Ontario and disruptive or intense daily pain in the WRHA. The top five indicators with the largest unadjusted difference between the two provinces were: the incidence of impaired locomotion within the home (28.4% higher in Ontario), the incidence of communication difficulty (12.7% higher in Ontario), the prevalence of hospitalization (11.0% higher in Ontario), the incidence of cognitive decline (10.9% in Ontario) and the prevalence of ADL/rehabilitation potential and not receiving rehabilitation therapies, which was 24.8% higher in the WRHA.

**Table 5:** Comparison of unadjusted and adjusted HCQI rates between the two provinces

	**Unadjusted rates**				*Adjusted rates		
	**Ontario**	**Winnipeg**	**Difference**	**p value**	**Ontario**	**Winnipeg**	**Difference**
	**%**				**%**		
**Prevalence HCQIs**	**n = 102,554**	**n = 9,250**			**n = 102,554**	**n = 9,250**	
ADL/rehabilitation potential and not receiving therapy	66.8	91.6	-24.8	<0.0001	-	-	-
Hospitalization	38.5	27.5	11.0	<0.0001	27.4	19.6	7.8
Disruptive or intense daily pain	35.5	35.0	0.5	0.29	35.6	35.7	-0.1
Falls	32.9	22.9	10.0	<0.0001	34.1	25.7	8.4
No Influenza vaccination	25.8	18.4	7.4	<0.0001	-	-	-
Social isolation	17.4	16.5	0.9	0.03	18.8	17.4	1.4
Negative mood	16.6	9.9	6.7	<0.0001	18.5	12.0	6.5
Inadequate pain control	16.4	19.4	-3.0	0.11	16.8	19.2	-2.4
Any injuries	9.9	14.0	-4.1	<0.0001	10.5	15.5	-5.0
Difficulty in locomotion and no assistive device	9.3	10.1	-0.8	<0.0001	25.2	22.9	2.3
Delirium	6.6	5.1	1.5	<0.0001	9.3	8.5	0.8
Weight loss	5.9	3.7	2.2	<0.0001	4.0	2.6	1.4
Inadequate meals	3.1	2.3	0.8	<0.0001	3.3	2.4	0.9
Neglect or abuse	3.1	1.0	2.1	<0.0001	3.8	1.3	2.5
Dehydration	1.4	0.3	1.1	<0.0001	0.8	0.2	0.6
No medication review by physician	1.1	1.8	-0.7	<0.0001	-	-	-
**Incidence HCQIs**	**(n = 14,999)**	**(n = 841)**			**(n = 14,999)**	**(n = 841)**	
Cognitive decline	76.3	65.4	10.9	<0.0001	76.3	67.9	8.4
ADL impairment	63.7	57.6	6.1	0.0004	65.0	61.7	3.3
Bladder incontinence	56.1	46.9	9.2	<0.0001	63.3	56.5	6.8
Communication difficulty	49.7	37.0	12.7	<0.0001	46.2	37.5	8.7
Impaired locomotion in home	44.3	15.9	28.4	<0.0001	53.8	22.2	31.6
Skin ulcers	8.8	3.6	5.2	<0.0001	5.5	2.4	3.1

*All risk-adjusted differences significant at p < 0.0001.

Of the 19 HCQIs that have client-level risk adjusters recommended, applying the risk adjustment resulted in a *decrease* in the difference between the adjusted rates across the majority (68.4%) of these indicators. One exception to this rule was the triggering rate for the incidence of locomotion decline in which the adjusted difference *increased* by 3.2% (i.e., from 28.4% to 31.6%). By and large, the differences between the risk adjusted rates were less than 10% between the two provinces.

## Discussion

Little is publicly known about home care clients in Canada, their needs, abilities and the quality of care that they receive. In this sample, over 90% of clients required full assistance with IADLs, about half had some level of cognitive impairment, 54% had daily pain that could be severe at times and 35% had ADL impairments, demonstrating the high level of need in this group. The quality indicators with the highest rates were related to clients having the potential to improve on their ADLs or having rehabilitation potential and not receiving appropriate therapies, cognitive decline, hospital use, disruptive daily pain and falls. Doran et al. [17] also used RAI-HC data to examine client safety in home care in both Ontario and in the Winnipeg area. Their measures of safety were not based on the interRAI HCQIs, but instead, focused on potential adverse events or safety issues that increase a person’s risk for an adverse event. Although not identical, there is significant overlap between their adverse events or safety risks and the definitions of the HCQIs (e.g., decline in physical function, history of falls, lack of medication review among clients taking multiple medications, hospital or emergency room visits). Similar to the current findings, they also found relatively high rates of issues such as hospital visits, emergency room visits and new falls. Several other recent publications have also focused on the area of patient safety in the home care sector [10,26,27]. However, the adverse events, defined here as issues that are likely a result of receiving health care, often do not closely align with the HCQIs used in the current study (e.g., unexpected death, development of a urinary tract infection, diabetic foot ulcer), making comparisons difficult or inappropriate.

In the current study, we also found that clients in Ontario were more likely to experience issues such as functional decline, cognitive decline and health instability when compared to clients in the WRHA despite being very similar on basic demographic characteristics. It was expected that Ontario would have higher HCQI rates based on the higher proportion of clients experiencing impairment on the health sub-scales and the higher rates on the CAPs and this was supported in the current analysis. Following risk adjustment, Ontario continued to have higher rates across nearly all of the HCQIs, although the magnitude of the difference was typically small, and was less than 10% in most cases. Similarly, Doran et al. [17] reported slightly higher rates, without using risk adjustment, for several safety risks (e.g., decline in physical function, history of two or more falls, decline in cognition) in Ontario than in Winnipeg, and again, these differences were typically less than 10%. Since the HCQIs represent *potential* quality issues, we cannot definitively conclude that Ontario was providing a sub-optimal level of care. Rather, the higher HCQI rates represent areas that may require further exploration to truly understand the process of care and other factors influencing these rates.

In contrast to this pattern, the ADL/rehabilitation potential and not receiving therapy HCQI showed a much higher rate in Winnipeg. This HCQI is made up of two parts: 1) the numerator, which includes clients who are not receiving physical therapy, occupational therapy or exercise therapy; and 2) the denominator which represents clients who trigger on the ADL CAP (i.e., clients who have the potential to benefit from rehabilitation or could improve their level of independence on ADLs). The fact that the WRHA had a higher rate on this QI could be due to their 14% lower rate on the CAP. Since the CAP makes up the denominator of the HCQI, as it decreases, the HCQI rate will go up, all other things being equal. Regardless of the driving force behind the differences observed in the current study, the sheer magnitude of the rate of this HCQI (91% in the WRHA and 67% in Ontario) warrants further exploration. A similarly high rate was also found across 11 European countries (range: 58.2%-98.6%) [9]. This indicator is a flag that we need to better understand the factors influencing eligibility for rehabilitation services in the community and consider what strategies could be implemented to improve the number of clients with access to these important services.

The HCQI provincial differences decreased for the majority of the HCQI rates following risk adjustment. In the instances where risk adjustment was applied and the provincial differences *increased*, the differences did not exceed 4%. This is very similar to earlier work by Dalby et al. [8]. Client-level risk adjustment appears to reduce the “extremes” in the rates, bringing both high and low rates closer to the centre. The fact that Ontario continued to have higher adjusted rates could indicate that other types of risk adjustment are warranted (e.g., agency-level risk adjustment) or it could reflect variations in processes of care that resulted in different outcomes between the two regions. However, the fact that most of the adjusted rates showed differences that were very small implies that there were likely no substantial variations in quality of care between these two regions. Determining how processes of care or additional risk adjustment might have influenced the rates was beyond the scope of the current study.

One interesting departure from previous research was our finding that the HCQI related to the incidence of impaired locomotion in the home had an adjusted difference that was 32% higher in Ontario. This is approximately 20% higher than what has been previously reported using RAI-HC data and the same methodology [8]. This may be a reflection of access to assistive devices to support locomotion in the home. Another similar HCQI, namely, the prevalence of difficulty in locomotion but not having an assistive device showed virtually no difference between the two regions, both before and following risk adjustment. So, it does not appear that the higher rate for the incidence HCQI can easily be explained by a lack of service provision for Ontario clients. It is possible that the risk adjusters for the incidence HCQI (e.g., difficulties with dressing, reduction in physical activity and cognitive impairment) are not adequately taking into account other factors that could increase this rate regardless of the quality of care. Further analysis may be warranted, at least for this particular indicator, to determine if other risk adjusters are relevant.

A limitation of this study was the inability to align the RAI-HC assessment data between the two regions. Only one year of complete data for the WRHA (2006–2007) was available, and data for this time frame were not available for Ontario. As such, we chose to use the most recent RAI-HC data in the two provinces. It is recognized that for an indicator like the prevalence of not receiving an influenza vaccination annual variations could be important. However, for the remaining indicators, it is unlikely that the rates would change dramatically from year to year. Given that the differences between the adjusted rates comparing Ontario and Winnipeg were small (typically differences of less than 10%), this provides some evidence that the timeframe discrepancy did not substantially influence the results.

This study was also limited by using only client-level risk adjustment. Agency-level risk adjustment has also been shown to have an influence on minimizing the HCQI rates between different regions [8]. Agency-level information such as characteristics of clients newly admitted into the home care program was not available through the existing database so an agency-level risk adjustment could not be completed. Caution must therefore be used when interpreting the results from this study because not all types of risk adjustment have been conducted. As such, there may still be other characteristics of the organization that could influence the HCQI rates. The entire area of risk adjustment for health care quality indicators is very new and little evidence exists to support one particular method over another [8,9].

Finally, the RAI-HC data are only available for long-stay clients, thereby introducing a possible selection bias. These clients may be more likely to experience functional and cognitive impairment, which could influence the HCQI rates. It is not appropriate to evaluate the quality of care for short-stay clients using the HCQIs as this could potentially penalize organizations who have had a very limited amount of time within which to assess the client and implement a care plan. As such, focusing on long-stay clients, when generating the HCQIs, is the most reasonable approach.

## Conclusions

This research utilized a near census of clients from Ontario and a very large sample of clients from the WRHA in order to bridge the gap in the home care literature with respect to understanding the needs of home care clients in Canada and the key quality issues identified with a set of HCQIs. Home care organizations can utilize the HCQIs to identify potential quality issues within their own region and also to compare their results to other parts of Canada utilizing the RAI-HC. As more regions begin to submit RAI-HC data to the Canadian Institute for Health Information, the potential will increase substantially for the HCQI rates to be generated for a number of provinces and territories and for these regions to begin to utilize their data in ways that support continuous quality improvement. The HCQIs represent a practical decision-support tool, collected at the point of care, that provide a means for home care providers and policy makers to assess and compare quality as part of the ongoing commitment to continually enhance the care provided in the community.

## Abbreviations

ADLs: Activities of Daily Living; HCQIs: Home Care Quality Indicators; IADLs: Instrumental ADLs; QI: Quality Indicator; RAI-HC: Resident Assessment Instrument for Home Care; WRHA: Winnipeg Regional Health Authority.

## Competing interests

The authors declare that they have no competing interests.

## Authors’ contributions

AM led the data analysis and took the lead on manuscript development. DG was involved in data analysis and preparation and review of the manuscript. Both authors read and approved the final manuscript.

## Pre-publication history

The pre-publication history for this paper can be accessed here:

http://www.biomedcentral.com/1472-6963/14/37/prepub
